# P-344. Nursing Home Rates of Healthcare-associated infections Requiring Hospitalization by Facility Characteristics and Staffing Levels, October 2021 – September 2022

**DOI:** 10.1093/ofid/ofae631.546

**Published:** 2025-01-29

**Authors:** Lindsey J Walker, Kelly M Hatfield, Sujan Reddy, Dustin Currie, Kara M Jacobs Slifka, Scott Fridkin, Heather N Jones, Andrea Rodriguez, Joseph D Lutgring

**Affiliations:** Centers for Disease Control and Prevention, Atlanta, Georgia; Centers for Disease Control and Prevention, Atlanta, Georgia; CDC, Atlanta, GA; Centers for Disease Control and Prevention, Atlanta, Georgia; Centers for Disease Control and Prevention, Atlanta, Georgia; Georgia Emerging Infections Program, Decatur, GA; Emory University School of Medicine, Atlanta, GA, Atlanta, Georgia; Centers for Disease Control and Prevention, Atlanta, Georgia; Centers for Disease Control and Prevention, Atlanta, Georgia; Division of Healthcare Quality Promotion, Centers for Disease Control and Prevention, Atlanta, GA

## Abstract

**Background:**

Healthcare-associated infections (HAIs) contribute to substantial morbidity among nursing home residents. The Centers for Medicare and Medicaid Services (CMS) tracks and reports a rate of HAIs requiring hospitalization per 100 resident stays (HAI rate) annually for nursing homes that is risk standardized by the health status and characteristics of the at-risk residents for each nursing home. We aimed to measure factors, including staffing, associated with nursing homes’ HAI rates.

**Figure 1.**
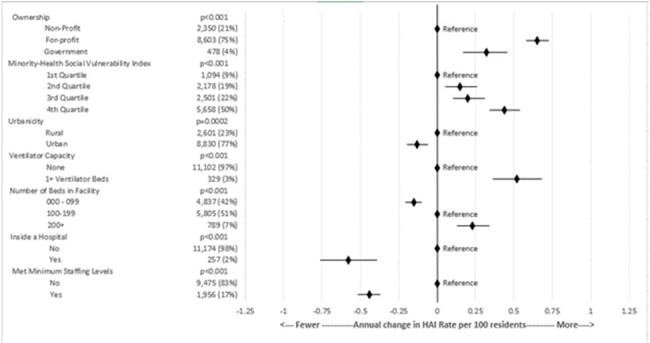
Description of nursing home characteristics among 11,431 nursing homes with a risk-standardized rate of healthcare-associated infections requiring hospitalization in fiscal year 2022. Estimated change in annual risk standardized rate of healthcare-associated infections requiring hospitalization per 100 residents (HAI rate) and p-values are calculated from parameter estimates in a multivariable linear regression model.

**Methods:**

Nursing home characteristics and the annual HAI rate from October 2021 – September 2022 were extracted from Nursing Home Compare. We described the distribution of nursing home characteristics including type of ownership, number of beds, urbanicity, ventilator care capability, location within hospital, and the minority health-social vulnerability index (MH-SVI) of the surrounding county. We quantified nursing homes as meeting minimum staffing standards if they reported ≥0.55 registered nurse hours per resident per day (HPRD), ≥2.45 nurse aide HPRD, and ≥3.48 total nurse staffing HPRD to reflect newly finalized CMS rules. Multivariable linear regression models were used to identify characteristics associated with the HAI rate.

**Results:**

The median annual HAI rate reported for 11,431 nursing homes was 6.79 hospitalizations per 100 residents (Q1-Q3: 5.93-7.87). In multivariable models, all characteristics were associated with the HAI rate (Figure 1). For-profit ownership was associated with 0.66 more infections per 100 residents (95% CI: 0.58, 0.73). Nursing homes located in counties in the highest MH-SVI quartile had 0.44 more HAIs per 100 residents per year (95% CI: 0.34, 0.54) than those in the lowest quartile. The 1,956 (17%) nursing homes that met minimum staffing standards had an estimated 0.44 fewer infections per 100 residents annually (95% CI: -0.52, -0.37) compared to nursing homes that did not, reflecting a 6% decrease in risk in an average facility.

**Conclusion:**

Meeting minimum staffing standards may reduce the rate of HAIs requiring hospitalization in nursing homes. Interventions and outreach efforts focused on increasing support for nursing homes in communities with high levels of vulnerability may also reduce inequities in the rate of HAIs which require hospitalization.

**Disclosures:**

**All Authors**: No reported disclosures

